# Supplementary material for: Probabilistic computing with NbOx metal-insulator transition-based self-oscillatory pbit
Source: Nat Commun. 2023 Nov 8;14:7199. doi: 10.1038/s41467-023-43085-6 (PMC10632392; doi:10.1038/s41467-023-43085-6)
Supplement: Supplementary file 1 — Supplementary Information [file 41467_2023_43085_MOESM1_ESM.pdf]

Supplementary Information

Probabilistic Computing with NbO_x Metal-Insulator Transition -based Self-oscillatory pbit

Hakseung Rhee[1], Gwangmin Kim[1], Hanchan Song[1], Woojoon Park[1], Do Hoon Kim[1], Jae Hyun In[1], Young Hyun Lee[1], and Kyung Min Kim[1,*]

[1]Department of Materials Science and Engineering, Korea Advanced Institute of Science and Technology (KAIST), Daejeon, 34141, Republic of Korea

*Correspondence and requests for materials should be addressed to K.M.K.
(Email: km.kim@kaist.ac.kr)

Contents

1. Area-dependent device capacitance and current characteristics
2. Dataset on probabilistic oscillation of NbO_x oscillator
3. Detailed description on measurement system's effect on device characterization
4. NbO_x oscillator simulation model parameters.
5. Electrical noise analysis on NbO_x oscillator
6. Simulation result for T_{MIT} as an oscillation generation threshold in NbO_x memristor
7. Probabilistic oscillation result of p-osc model
8. Derivation of general solution for the simultaneous equations of the Ornstein-Uhlenbeck process of the NbO_x oscillator
9. In-depth description of the MBM to solving MVC problem
10. Brute-force algorithm for minimum vertex cover problem
11. Autocorrelation function to all V_{ext} conditions for experimental NbO_x oscillator
12. Energy heatmap and detailed explanation of Y_{cor} and $\bar{Y}_{incor}$ in the MBM system
13. Experiment on probabilistic oscillation in NbO_x oscillator fabricated with different condition

1. Area-dependent device capacitance and current characteristics

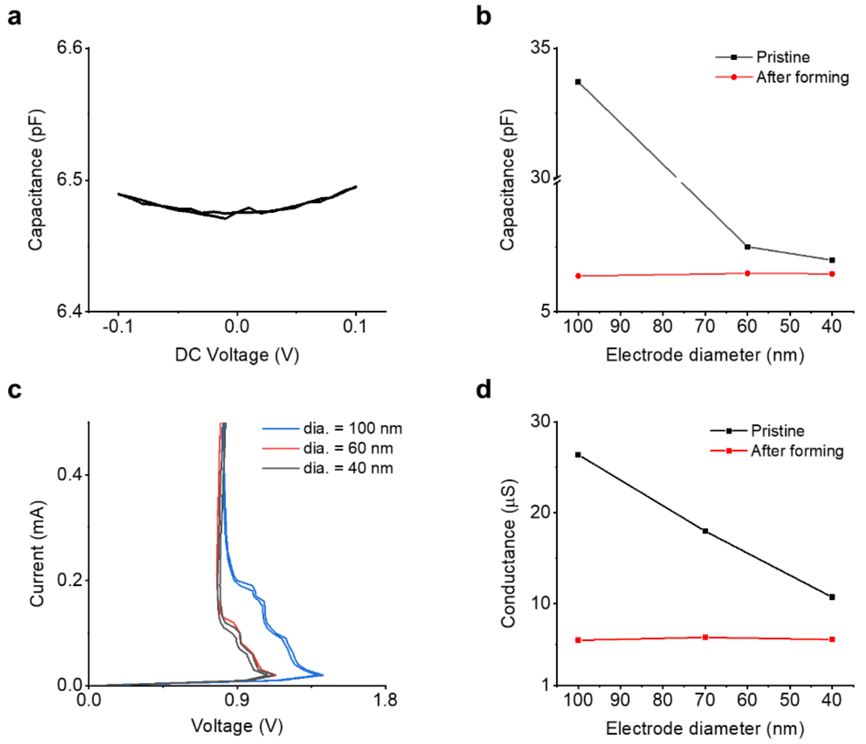

Fig. S1. a *C*-*V* measurement result of NbO_x memristor with diameter of 40 nm. **b** Capacitances of NbO_x memristors with different diameter (100 nm, 60 nm, 40 nm) before (pristine) and after elecroforming. **c** NDR behaviors with different diameter (100 nm, 60 nm, 40 nm) after electroforming. They exhibited almost identical NDR behaviors considering the area variation, especially at the off state. **d** Device conductances before (pristine) and after elecroforming at the off state read (at -0.1 V). These results strongy support the core-shell model, where the localized core dominates the NDR behaivors. Some variations in NDR behaviors were noted, likely attributable to shell characteristics, including shell area and conductivity.

2. Dataset on probabilistic oscillation of NbO_x oscillator

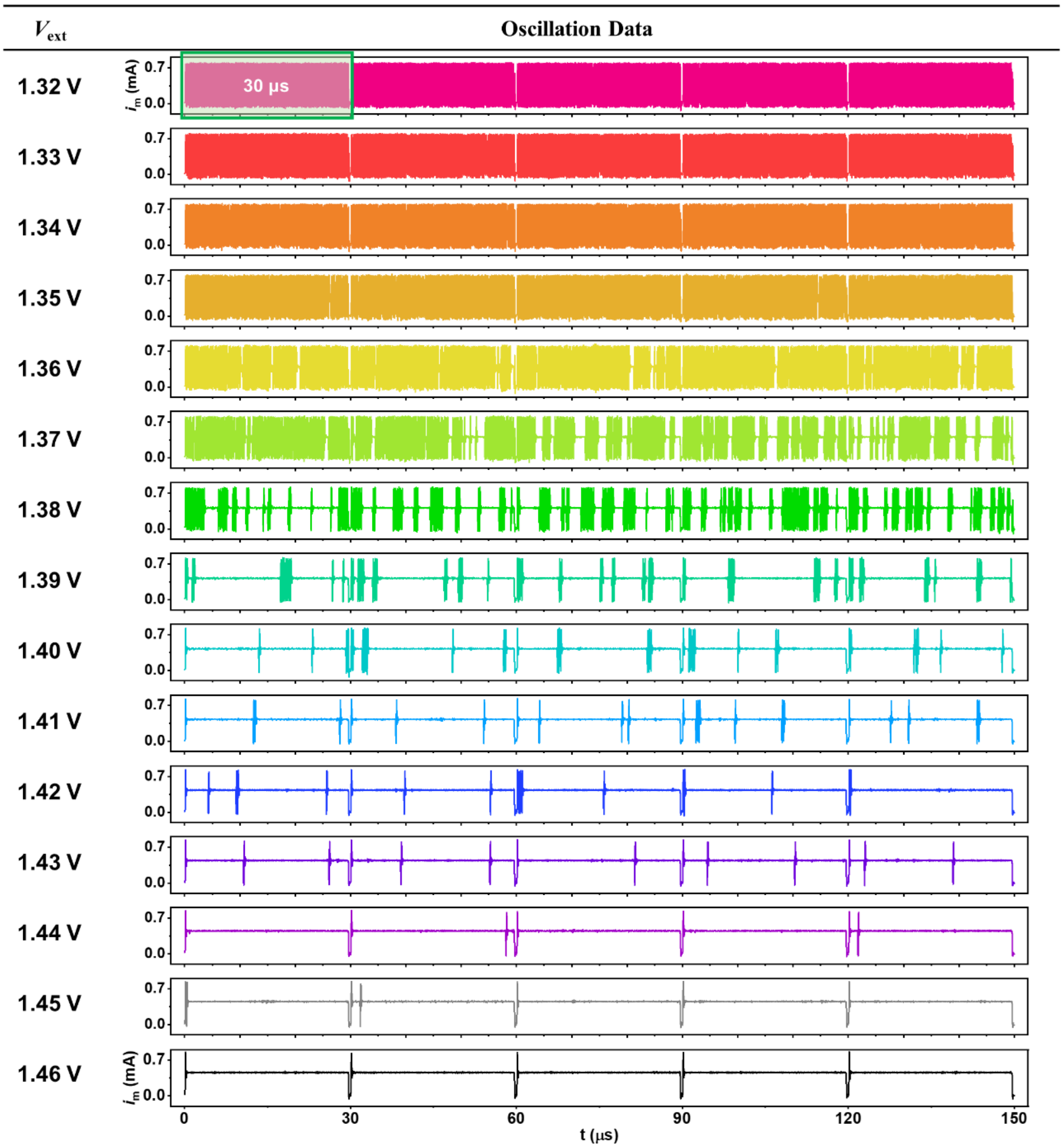

Fig. S2. Probabilistic oscillation dataset of NbO_x oscillator.

Five tests were performed for each external voltage (V_{ext}) condition. For each test, the current oscillations were measured under 30 µs width of a single V_{ext} pulse. The data collection process for V_{ext} cases involved collecting data once for all V_{ext} cases initially and then repeating the process four more times for each V_{ext} case individually.

3. Detailed description of the measurement system's effect on device characterization

a

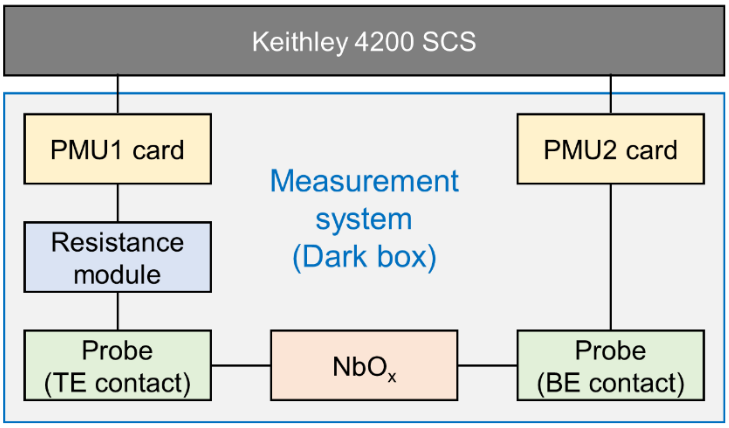

b

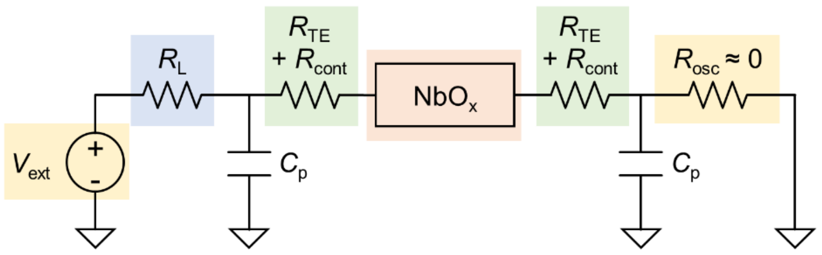

Fig. S3. NbO_x oscillator measurement system. a A schematic of measurement system with Keithley 4200 SCS semiconductor analyzer and dark box. **b** Equivalent circuit diagram of **a**

The measurement system we used to evaluate the NbO_x oscillator is illustrated in Fig. S3a. Two PMU cards connected to a Keithley 4200 SCS semiconductor analyzer apply voltage to both terminals of the NbO_x memristor and read the output. PMU 1 is connected to a series resistor module, and the probe makes contact with the top electrode of the NbO_x memristor. Another probe contacts the bottom electrode and is directly connected to PMU 2. All components, except for the semiconductor analyzer, are located within a dark box.

Based on this setup, an equivalent NbO_x oscillator circuit is presented in Fig. S3b. Circuit elements corresponding to components in the measurement system are colored to match those in Fig. S3a.

	R_L	R_{TE} + R_{cont}	R_{BE} + R_{cont}	R_{osc}	C_p	R_{total}	V_{ext}	Oscillate?
**Unit**	Ω	Ω	Ω	Ω	pF	Ω	V	-
**Case 1 (Reference)**	1200	0	0	0	0	1200	1.32	Yes
							1.33	Yes
**Case 2**	1200	0	0	0	20	1200	1.32	Yes
							1.33	No
**Case 3**	1100	50	50	0	20	1200	1.32	No
							1.33	No

Table S1. Simulation parameters of NbO_x oscillator measurement system

We used a noise-free NbO_x memristor model for the simulation, and the results under various conditions are summarized in Table S1.

For case 1, we configured the oscillator circuit identically to the one described in the main text, setting only the load resistor (R_L) to 1200 Ω and removing all other elements. When a DC voltage (V_{ext}) of 1.32 V and 1.33 V was applied, we observed oscillations in the output current, consistent with Fig. 2d in the main text.

In case 2, we introduced a parasitic capacitance (C_p) to reflect the electrically isolated state of the probe in the measurement system. As a result, the node voltage at the front end of the NbO_x device becomes time-dependent due to C_p. Under these conditions, we observed oscillations at V_{ext} = 1.32 V but not at 1.33 V, despite the total series resistance being the same 1200 Ω as in case 1. This indicates that C_p influences the oscillation conditions of the NbO_x oscillator. In particular, it can be seen that the non-oscillation of V_{ext} = 1.33 V, despite the circuit being configured with the same series resistor, is entirely an effect of C_p compared to case 1.

This trend is even more severe in case 3, which incorporates the resistances of the top and bottom electrodes as well as the contact resistance of the probe, thus closely mimicking the actual experimental setup. In this case, the circuit did not oscillate even at V_{ext} = 1.32 V, so it can be seen that the effect of voltage dividing due to the series resistances further increases the influence of the C_p on oscillation.

From these comparative simulation results, in a real-world experimental setup, the presence of C_p has the effect of making the system to converge rather than oscillate from a lower point than when calculated considering only the equilibrium (static) state ($i_L = i_{osc} = \frac{V_{ext}-V_m}{R_L}$) of the system. Consequently, even though the equilibrium point is formed at a point slightly below the NDR-2 region, as shown in the red curves in Figure 1a, it can be understood that probabilistic oscillations can occur in the measurement environment due to the resulting non-oscillatory state caused by C_p.

4. NbO_x oscillator simulation model parameters.

Parameter	Value	Units
d	15.0E-9	m
A	12.56E-16	m^2
q	1.60E-19	C
k_B	8.617E-5	eV/K
σ_0	8.40E+4	S/m
E_a	0.255	eV
C_{th}	5.0E-17	Ws/K
$R_{th.I}$	1.70E+6	K/W
$R_{th.M}$	2.20E+6	K/W
p	100	-
T_{amb}	300	K
T_{MIT}	1070	K

Table S2. Parameters used in NbO_x oscillator model.

5. Electrical noise analysis on NbO_x oscillator

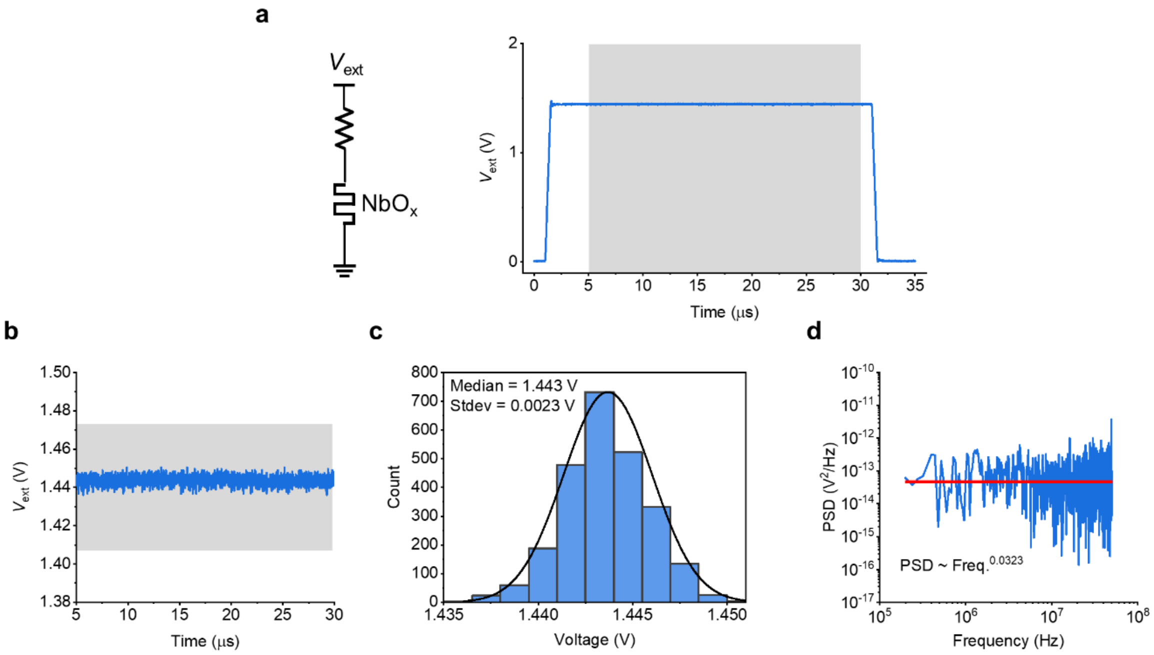

Fig. S4. **a** Circuit diagram of NbO_x oscillator and measured applied external voltage (V_{ext}) to time in case of V_{ext} = 1.46 V. **b** V_{ext} vs time plot magnifying the region from 5 us to 30 us (gray-colored region in **a**). **c** Histogram of V_{ext}. **d** Power spectral density (PSD) plot of V_{ext} to frequency, showing the relationship between PSD and frequency, indicating that the closer a frequency is to zero, the closer it is to white noise.

6. Simulation result for T_{MIT} as an oscillation generation threshold in NbO_x memristor

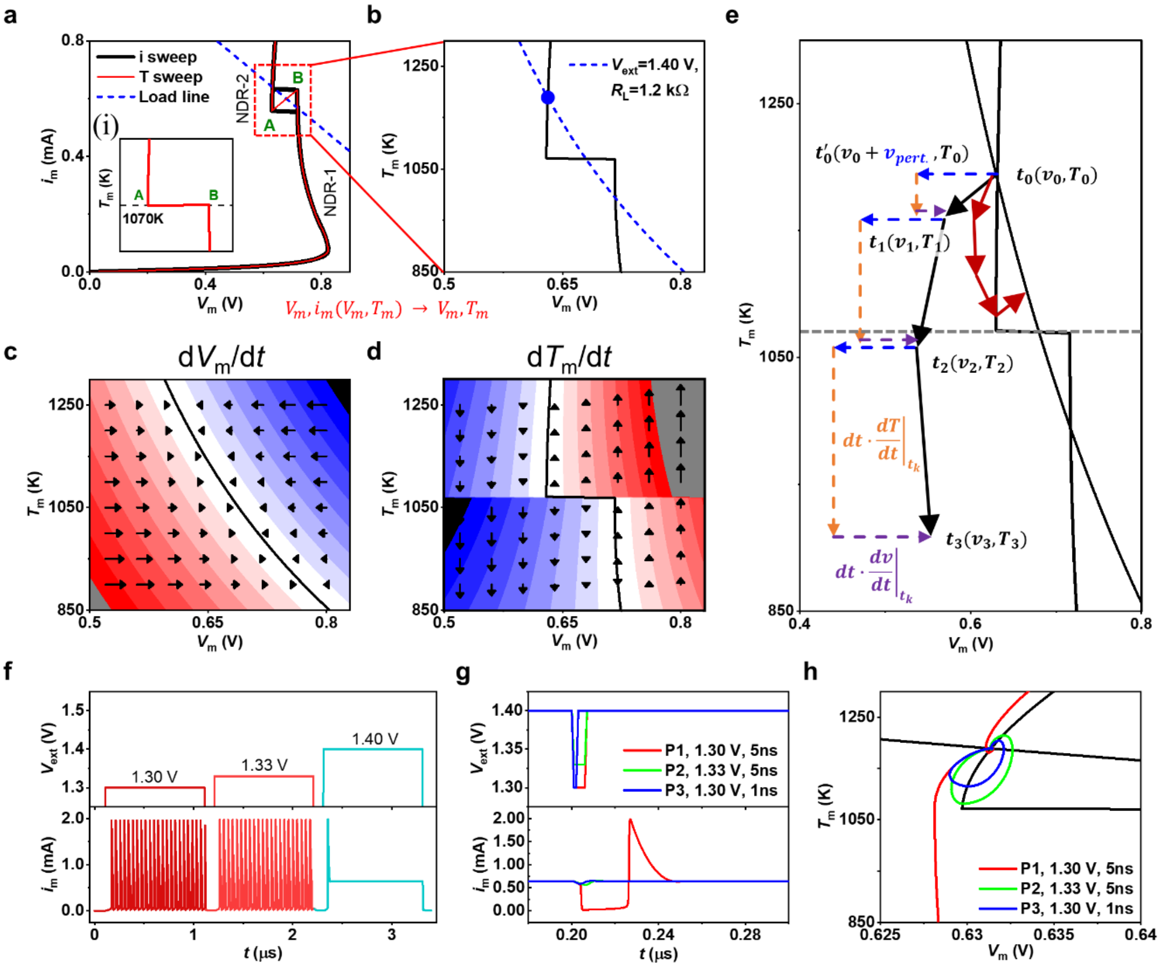

Fig. S5. Details on the noise-driven oscillation in NbO_x oscillator model. a Simulation *I-V* curve by the current sweep (black) and temperature sweep (red), and load line (blue dotted) at V_{ext} = 1.40 V, R_L = 1.2 kΩ. (i) The inset is the corresponding *T-V* curve. **b** The magnified *T-V* curve around the NDR-2 and the load line. **c, d** dv_m/dt and dT_m/dt vector maps around the NDR-2. **e** The pseudo-equilibrium state trajectories (black and red vectors) in the *T-V* space by the negative voltage perturbation (blue vector) from the equilibrium state. The purple and yellow vectors represent the dv_m/dt and dT_m/dt, respectively**.** **f** The simulated output currents at V_{ext} = 1.30 V, 1.33 V, and 1.40 V from the numerical model. **g** The input voltages and corresponding output currents at various perturbating conditions (P1-P3). **h** The pseudo-equilibrium state trajectories near NDR-2 for P1-P3.

Fig. S5a shows the equilibrium *I-V* curve (black) obtained from the numerical model by Equations 1-4, where $dT_m/dt = 0$ and $dv_m/dt = 0$. The blue dashed line shows the load line by R_L = 1.2 kΩ at V_{ext} = 1.40 V. Fig. S5b enlarges the *T-V* curve near the metal-insulator transition region. At the given load line, the operating point (blue dot) is at the positive differential resistance region above the NDR-2 region, so the self-oscillator is not oscillating but stays at the on-state.

At the equilibrium state, the perturbation may change the operating point to the metastable space.[1,2] Once it happens, the time-dependent temperature gradient (dT_m/dt) and voltage gradient (dv_m/dt) at the point act as a restoring force that drives it to an equilibrium point. Fig. S5c-d show the calculated dv_m/dt and dT_m/dt vectors on the *T-V* space, respectively. The black lines are equilibrium lines giving dv_m/dt and $dT_m/dt = 0$, which are equal to the load line and equilibrium *T-V* curve. In Fig. S5d, the dT_m/dt was abruptly changed at the metal-insulator transition temperature (T_{MIT}) due to the drastic change of R_{th}

between the metallic phase ($R_{th,M}$) and the insulating phase ($R_{th,I}$). By using the two gradient vectors, the trajectory from the metastable state to the equilibrium state over time can be estimated. Fig. S5e shows the schematics of two perturbation results in a completely different trajectories; one is a larger voltage perturbation case (black arrow), and the other is a smaller voltage perturbation case (red arrow). The $t_0'(v_0, T_0)$ is the equilibrium point which is defined by the intersection of dv_m/dt and $dT_m/dt = 0$ at a given V_{ext}. When external voltage perturbation (v_{pert}, blue vector) is engaged to t_0, it tends to go to $t_0'(v_0 + v_{pert}, T_0)$. However, due to the restore forces (yellow vector), the metastable state after dt can be defined by $t_1(v_1, T_1) = t_1\left(v_0 + v_{pert} + \left.\frac{dv}{dt}\right|_{t_0'} dt, T_0 + \left.\frac{dT}{dt}\right|_{t_0'} dt\right)$ (black vector). As such, the sum of perturbation and restore forces gives the metastable points trajectory toward the equilibrium point over time. This results in a spiral $t_n(v_n, T_n)$ trajectory at the T-V space. Here, when the perturbation was small, the metastable state temperature was kept higher than the T_{MIT} and it eventually converged to the initial equilibrium state (red trajectory). Whereas, when the perturbation was large, the metastable temperature dropped below the T_{MIT}, so the temperature gradient drastically increased negatively due to the metallic-to-insulator transition. This resulted in a cooling down of the NbO_x device to the off-state (black trajectory). Then, due to the positive voltage gradient at the off-state, the v increased until it exceeded the V_{th}, and the device returned to the on-state, generating an oscillation peak. Then, it converged to the initial equilibrium state. As a result, perturbation-induced oscillation could be possible when the perturbation was sufficient. In addition to voltage perturbation, temperature perturbation can be considered similarly.

Figs. S5f-h show the perturbation-induced oscillation generation by intentionally applying a perturbing pulse in the numerical simulation model. Before that, we investigated the boundary condition of V_{ext} between the periodic oscillation and no oscillation as shown in Fig. S5f. The model confirmed that the V_{ext} under 1.33 V generated the periodic oscillation, and over 1.40 V showed no oscillation at the on-state. Next, we set the device to the on-state (V_{ext} = 1.40 V) and then inserted the following three perturbing pulses: 1.30 V for 5 ns (P1, red), 1.33 V for 5 ns (P2, green), and 1.30 V for 1 ns (P3, blue) as shown in the top panel of Fig. S5g. The lower panel shows the output currents for three perturbating cases. Despite all voltage conditions corresponding to the oscillation condition, only P1 generated an oscillation. Fig. S5h shows the $t_n(v_n, T_n)$ trajectory of the three cases. The perturbation by P1 was sufficiently large to cause the temperature to go down below the T_{MIT}. Whereas the perturbation by P2 and P3 was weak, making the non-equilibrium state converge to the equilibrium point via a small spiral trajectory. Thus, these simulation results show that T_{MIT} corresponds to the threshold for the occurrence of oscillation generation for the metastable state of the NbO_x oscillator.

7. Probabilistic oscillation result of p-osc model

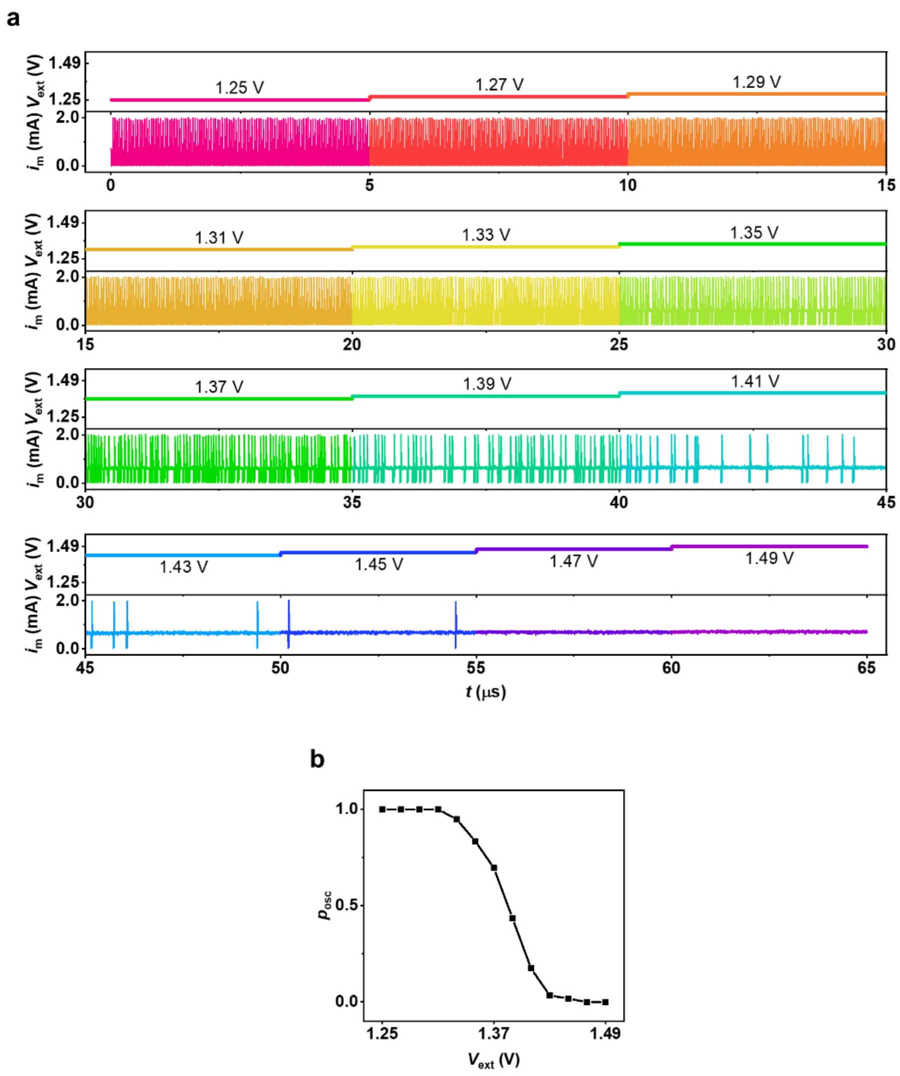

Fig. S6. a Probabilistic oscillation result of the numerical oscillator model with the OU process in the same case of Fig. 3 in main text as increasing the V_{ext} from 1.25 V to 1.49 V with a 0.02 V interval. **b** Oscillation probability distribution calculated from the above oscillation results.

8. Derivation of general solution for the simultaneous equations of the OU process of the NbO_x oscillator

The two stochastic differential equations (SDE) of the Ornstein-Uhlenbeck (OU) process are given as Equations 5 and 6 in main text:

Equation 5: $$dT(t) = \frac{1}{R_{th}C_{th}}\left(R_{th}iv(t) + T_{amb} - T(t)\right)dt + \sigma_T dW(t) \quad \text{(S1)}$$

Equation 6: $$dv(t) = \frac{1}{R_L C_m}\left(v_{ext} - R_L i - v(t)\right)dt + \sigma_v dW(t) \quad \text{(S2)}$$

, which consists of the drift term and diffusion term.

From the simulation results of Supplementary Information section 6, we conclude that oscillation is generated due to the drastic acceleration at T_{MIT}. Thus the temperature threshold (T_{MIT}) is assumed as a boundary condition for oscillation generation. By solving the simultaneous equations of Equations S1 and S2, we derived the temperature variation due to collective interference by temperature and voltage noises. This approximated calculation is based on linear dependence and assumes stationarity and no feedback.

First, we derive the general solution of Equation S2, the stochastic process of voltage, as follows.

$$dv(t) + \frac{1}{R_L C_m}v(t)dt = \frac{1}{R_L C_m}(v_{ext} - R_L i)dt + \sigma_v dW(t) \quad \text{(S3)}$$

By multiplying $e^{\frac{t}{R_L C_m}}$ to both sides,

$$e^{\frac{t}{R_L C_m}}dv(t) + e^{\frac{t}{R_L C_m}}\frac{1}{R_L C_m}v(t)dt = e^{\frac{t}{R_L C_m}}\frac{1}{R_L C_m}(v_{ext} - R_L i)dt + e^{\frac{t}{R_L C_m}}\sigma_v dW(t) \quad \text{(S4)}$$

Equation S4 is equal to Equation S5 as below:

$$\frac{d}{dt}(e^{\frac{t}{R_L C_m}}v(t)) = e^{\frac{t}{R_L C_m}}\frac{1}{R_L C_m}(v_{ext} - R_L i) + e^{\frac{t}{R_L C_m}}\sigma_v\frac{dW(t)}{dt} \quad \text{(S5)}$$

By integrating both sides with respect to time from 0 to $t^{'}$

$$d(e^{\frac{t}{R_L C_m}}v(t)) = e^{\frac{t}{R_L C_m}}\frac{1}{R_L C_m}(v_{ext} - R_L i)dt + e^{\frac{t}{R_L C_m}}\sigma_v dW(t) \quad \text{(S6)}$$

$$\int_0^{t^{'}}(e^{\frac{t}{R_L C_m}}v(t)) = \int_0^{t^{'}} e^{\frac{t}{R_L C_m}}\frac{1}{R_L C_m}(v_{ext} - R_L i)dt + e^{\frac{t}{R_L C_m}}\sigma_v dW(t) \quad \text{(S7)}$$

$$e^{\frac{t^{'}}{R_L C_m}}v(T) - v(0) = (v_{ext} - R_L i)\left(e^{\frac{t^{'}}{R_L C_m}} - 1\right) + \sigma_v\int_0^{t^{'}} e^{\frac{t}{R_L C_m}}dW(t) \quad \text{(S8)}$$

$$v\left(t^{'}\right) = v(0)e^{-\frac{t^{'}}{R_L C_m}} + (v_{ext} - R_L i)\left(1 - e^{-\frac{t^{'}}{R_L C_m}}\right) + \sigma_v\int_0^{t^{'}} e^{-\frac{t^{'}-t}{R_L C_m}}dW(t) \quad \text{(S9)}$$

From Equation S9, we can calculate the probability distribution. Because Wiener process is the definite integral of a white noise Gaussian process, it is characterized by having zero mean, unit variance[3,4]. Then the mean and variance of Equation S9 are calculated as below:

$$\mathbb{E}[v(t)] = v(0)e^{-\frac{t'}{R_L C_m}} + (v_{ext} - R_L i)\left(1 - e^{-\frac{t'}{R_L C_m}}\right) \quad \text{(S10)}$$

$$\sigma^2[v(t)] = \mathbb{E}[(v(t) - \mathbb{E}[v(t)])^2] = \frac{R_L C_m \sigma_v^2}{2}(1 - e^{\frac{2t'}{R_L C_m}}) \quad \text{(S11)}$$

From these equations, the long-term mean and variance of voltage distribution are

$$\lim_{t' \to \infty} \mathbb{E}[v(t)] = v_{ext} - R_L i \quad \text{(S12)}$$

$$\lim_{t' \to \infty} \sigma^2[v(t)] = \frac{R_L C_m \sigma_v^2}{2} \quad \text{(S13)}$$

Next, we calculate the temperature distribution induced by collective interference of temperature and voltage by inserting the result of voltage variation into the voltage term in the OU process equation of temperature.

$$dT(t) = \frac{1}{R_{th} C_{th}}\left(\left(R_{th} i v(t) + \left.\frac{dT}{dv}\right|_v \Delta v + T_{amb}\right) - T(t)\right) dt + \sigma_T dW(t) \quad \text{(S14)}$$

Thereafter, we assigned the voltage variation term (Δv) as $\Delta v = \epsilon \cdot \sigma[v(t)] = \epsilon \cdot \sigma_v \sqrt{\frac{R_L C_m}{2}}$, where ϵ has a standard normal distribution $\mathcal{N}(0,\ 1)$. As a consequence, the temperature variation induced by voltage noise in infinitesimal time interval dt is described as below equation.

$$\frac{1}{R_{th} C_{th}}\left(\left.\frac{dT}{dv}\right|_v \Delta v\right) dt = \frac{1}{R_{th} C_{th}}\left(\left.\frac{dT}{dv}\right|_v \sigma_v \sqrt{\frac{R_L C_m}{2}}\right) \epsilon \cdot dt \quad \text{(S15)}$$

In addition, the Wiener process is defined as a Markov process with zero mean and variance of Δt, thus a change during a small period of time dt, $dW(t)$, is equal to $\epsilon\sqrt{dt}$. Then, Equation S15 can be modified as below:

$$\frac{1}{R_{th} C_{th}}\left(\left.\frac{dT}{dv}\right|_v \sigma_v \sqrt{\frac{R_L C_m}{2}}\right) \epsilon \cdot \sqrt{dt} \cdot \sqrt{dt} = \frac{1}{R_{th} C_{th}}\left(\left.\frac{dT}{dv}\right|_v \sigma_v \sqrt{\frac{R_L C_m}{2}}\right) \sqrt{dt} \cdot dW(t) \quad \text{(S16)}$$

As a result, the temperature variation induced by voltage noise can be included in the noise term, and the stochastic differential equation of temperature considering collective noises of temperature and voltage is described in the following equation.

$$\begin{aligned} dT(t) = &\frac{1}{R_{th} C_{th}}\big(R_{th} i v(t) + T_{amb} - T(t)\big) dt \\ &+ \left(\sigma_T + \frac{1}{R_{th} C_{th}} \left.\frac{dT}{dv}\right|_v \sigma_v \sqrt{\frac{R_L C_m \Delta t}{2}}\right) dW(t) \end{aligned} \quad \text{(S17)}$$

In the same way of calculating the mean (Equation S12) and variance (Equation S13) from Equation S2, the long-term mean and variance of Equation S17 can be calculated as below:

$$\lim_{t' \to \infty} \mathbb{E}[T(t)] = R_{th} i v(t) + T_{amb} \quad \text{(S18)}$$

$$\lim_{t' \to \infty} \sigma[T(t)] = \sqrt{\frac{R_{th}C_{th}}{2}}\left(\sigma_T + \frac{1}{R_{th}C_{th}} \cdot \frac{dT}{dv}\bigg|_v \cdot \sigma_v \sqrt{\frac{R_L C_m \Delta t}{2}}\right) \qquad \text{(S19)}$$

which are Equations 7 and 8 in main text. The correspondence between this derived general solution and the numerical model (p-osc model) is illustrated as below.

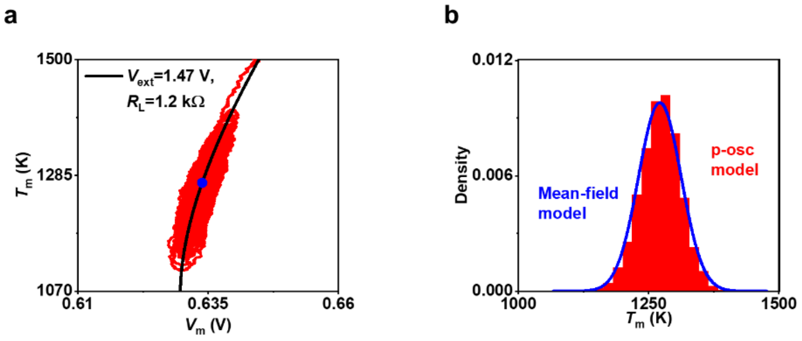

Fig. S7. a *T*-*V* plot of pseudo-equilibrium states of numerical oscillator model at V_{ext} = 1.47 V, R_L = 1.2 kΩ (blue dot is an equilibrium point). **b** Temperature histogram (red) from *T*-*V* plot and normal distribution (blue) from compact stochastic oscillator model (Equations 7 and 8) under the same condition.

9. In-depth description of the MBM to solving MVC problem

As described in the manuscript, in the operation of the Boltzmann machine, the negative derivative of the Hamiltonian ($h(\mathbf{X})$) for each pbit at the current time step ($D_i^t = -\partial h(\mathbf{X}^t)/\partial X_i$) is used as the input value for that pbit at the next time step. However, as can be seen from the $h(\mathbf{X})$ in Equation 10 of main text, D_i^t depends on the absolute size of the $h(\mathbf{X})$ which is affected by α and β (or additional parameters in other problems[5]) or the way the developed $h(\mathbf{X})$ is defined. Therefore, when simply using D_i^t as an input value for the next step, an excessively large or small D_i^t may make the stochastic behavior too deterministic, or may not induce significant differences in others. Thus, to mitigate this problem previously, we arithmetically calculated the maximum and minimum values of D_i^t that could be obtained from the initially set $h(\mathbf{X})$ and normalized D_i^t into the device operating range V_{ext}.

In case of minimum vertex covering (MVC) problem[5], $h(\mathbf{X})$ as quadratic unconstrained binary optimization (QUBO) formulation is described as Equation S20 (same as Equation 10 of main text), where $X_v \in \{0, 1\}$.

$$h(\mathbf{X}) = \alpha \sum_{u,v \in E} (1 - X_u)(1 - X_v) + \beta \sum_{v \in V} X_v \tag{S20}$$

If we rewrite this equation in vector-matrix form, $h(\mathbf{X})$ is described as Equation S21.

$$h(\mathbf{X}) = \alpha \mathbf{X}^T (0.5A - D)\mathbf{X} + \alpha \mathbb{J}^T (0.5A)\mathbb{J} + \beta \mathbf{X}^T \mathbf{X} \tag{S21}$$

$$h(\vec{X}) = \mathbf{X}^T \{\alpha(0.5A - D) + \beta I\} \mathbf{X} + 2\alpha \tag{S22}$$

where A is adjacency matrix, D is degree matrix, $\mathbb{J}$ is all-ones matrix of the same size as A and D, and I is identity matrix. If we set each element in $(0.5A - D)$ as P_{ij}, Equation S22 is describes as below:

$$h(\mathbf{X}) = \mathbf{X}^T \begin{pmatrix} \alpha P_{11} + \beta & \alpha P_{12} & \cdots & \alpha P_{1n} \\ \alpha P_{21} & \alpha P_{22} + \beta & \cdots & \alpha P_{2n} \\ \vdots & \vdots & \ddots & \vdots \\ \alpha P_{n1} & \alpha P_{n2} & \cdots & \alpha P_{nn} + \beta \end{pmatrix} \mathbf{X} + 2\alpha \tag{S23}$$

Due the symmetric property of adjacency matrix A,

$$h(\mathbf{X}) = \mathbf{X}^T \begin{pmatrix} \alpha P_{11} + \beta & \alpha P_{12} & \cdots & \alpha P_{1n} \\ \alpha P_{12} & \alpha P_{22} + \beta & \cdots & \alpha P_{2n} \\ \vdots & \vdots & \ddots & \vdots \\ \alpha P_{1n} & \alpha P_{2n} & \cdots & \alpha P_{nn} + \beta \end{pmatrix} \mathbf{X} + 2\alpha = \mathbf{X}^T Q \mathbf{X} + 2\alpha \tag{S24}$$

While calculation of $h(\mathbf{X})$, $X_v^2 = X_v$ due to $X_v \in \{0, 1\}$. Thus, $\partial h(\mathbf{X}^t)/\partial X_i$ can be simply described as below equation, which aligns with neuronal dynamics between neurons[6].

$$\frac{\partial h(\mathbf{X})}{\partial X_i} = Q_{ii} + \sum_{j=1, j\neq i}^{n} 2Q_{ij} X_j \tag{S25}$$

The off-diagonal elements of Q is equal to corresponding element of $0.5A$ and the adjacency matrix A has only positive value. Thus, the input value of the ith pbit for the next time step can have a range of values as below:

$$-\frac{\partial h(\mathbf{X})}{\partial X_i} \in \left[-Q_{ii}, -\left(Q_{ii} + \sum_{j=1, j\neq i}^{n} 2Q_{ij} \right) \right] \tag{S26}$$

So, from the final Equation S26, we can get n sets of maximum and minimum values of $-\frac{\partial h(\mathbf{X})}{\partial X_i}$ for n pbit systems. We then take the absolute values of all n sets and designated the largest absolute value among the $2n$ values as D_{max} and D_{min} = -D_{max}.

In case of graph G(6, 7) in Fig. 3b, the $A, D,$ and Q matrix is defined as below.

$$A = \begin{pmatrix} 0 & 1 & 0 & 0 & 0 & 1 \\ 1 & 0 & 1 & 0 & 0 & 1 \\ 0 & 1 & 0 & 1 & 1 & 0 \\ 0 & 0 & 1 & 0 & 0 & 0 \\ 0 & 0 & 1 & 0 & 0 & 1 \\ 1 & 1 & 0 & 0 & 1 & 0 \end{pmatrix}, D = \begin{pmatrix} 2 & 0 & 0 & 0 & 0 & 0 \\ 0 & 3 & 0 & 0 & 0 & 0 \\ 0 & 0 & 3 & 0 & 0 & 0 \\ 0 & 0 & 0 & 1 & 0 & 0 \\ 0 & 0 & 0 & 0 & 2 & 0 \\ 0 & 0 & 0 & 0 & 0 & 3 \end{pmatrix},$$

$$Q = \begin{pmatrix} \beta - 2\alpha & 0.5\alpha & 0 & 0 & 0 & 0.5\alpha \\ 0.5\alpha & \beta - 3\alpha & 0.5\alpha & 0 & 0 & 0.5\alpha \\ 0 & 0.5\alpha & \beta - 3\alpha & 0.5\alpha & 0.5\alpha & 0 \\ 0 & 0 & 0.5\alpha & \beta - \alpha & 0 & 0 \\ 0 & 0 & 0.5\alpha & 0 & \beta - 2\alpha & 0.5\alpha \\ 0.5\alpha & 0.5\alpha & 0 & 0 & 0.5\alpha & \beta - 3\alpha \end{pmatrix} \tag{S27}$$

Then, the minimum, maximum value set for each pbit can be derived as below equations. We have set α = 2 and β = 1 in this research.

$$-\frac{\partial h(\mathbf{X})}{\partial X_1} \in [-1,3] \tag{S28}$$

$$-\frac{\partial h(\mathbf{X})}{\partial X_2} \in [-1,5] \tag{S29}$$

$$-\frac{\partial h(\mathbf{X})}{\partial X_3} \in [-1,5] \tag{S30}$$

$$-\frac{\partial h(\mathbf{X})}{\partial X_4} \in [-1,1] \tag{S31}$$

$$-\frac{\partial h(\mathbf{X})}{\partial X_5} \in [-1,3] \tag{S32}$$

$$-\frac{\partial h(\mathbf{X})}{\partial X_6} \in [-1,5] \tag{S33}$$

Thus, D_{max} and D_{min} are designated as 5 and -5 in this case.

10. Brute-force algorithm for minimum vertex cover problem

The brute-force algorithm for solving the MVC problem proceeds as follows:

1. Initialize: For a graph *G* composed of *n* vertices and *m* edges, create a matrix *V* of length *n*.

2. Generate subset: Produce all possible subsets *v* of *V*.

3. Check for vertex cover: For each subset *v* of *V*, perform the following steps until all subsets have been examined:

3-1. Determine if *v* is a vertex cover by checking if every edge (*u*, *v*) in *E* is incident to at least one vertex in *v*.

3-2. If *v* is a vertex cover, compare its size (the sum of all elements in *v*) to the size of the current smallest vertex cover.

3-3. Update the minimum vertex cover to *v* if its size is smaller.

4. Return result: Return the minimum vertex cover.

Its pseudo code is shown below:

Algorithm S1: Brute-force algorithm for minimum vertex cover problem

```
// Input: Graph G with n vertices and m edges
// Output: Minimum vertex cover. min_vertex_cover

// Initialize
min_vertex_cover = {}  // Empty set
min_size = INF  // Initialize to a value greater than the maximum possible size (INF is infinity)
V = [V1, V2, ..., Vn]  // List of vertices

// Generate Subsets
all_subsets = PowerSet(V)  // Generate all subsets of V

// Check for Vertex Cover
for each subset v in all_subsets:
    is_vertex_cover = True

    // Check if v is a vertex cover
    for each edge (u, w) in G.edges:
        if u not in v and w not in v:
            is_vertex_cover = False
            break

    // Compare its size to the current smallest vertex cover
    if is_vertex_cover:
        size_v = len(v)

        if size_v < min_size:
            min_size = size_v
            min_vertex_cover = v  // Update the minimum vertex cover

// Return Result
return min_vertex_cover
```

11. Autocorrelation function to all V_{ext} conditions for experimental NbO_x oscillator

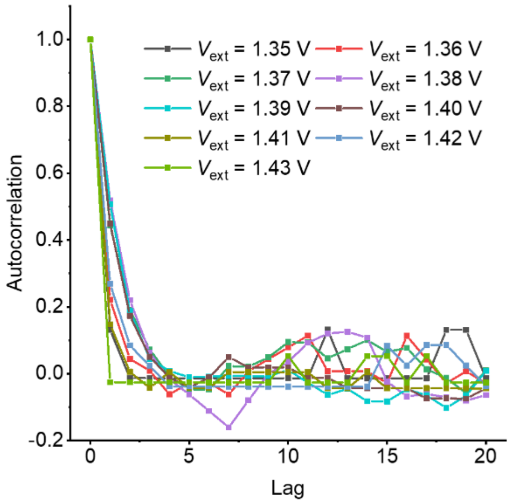

Fig. S8. Autocorrelation function of all V_{ext} conditions of experimental NbO_x oscillator.

12. Energy heatmap and detailed explanation of $\mathbf{Y}_{\mathbf{cor}}$ and $\bar{\mathbf{Y}}_{\mathbf{incor}}$ in the MBM system

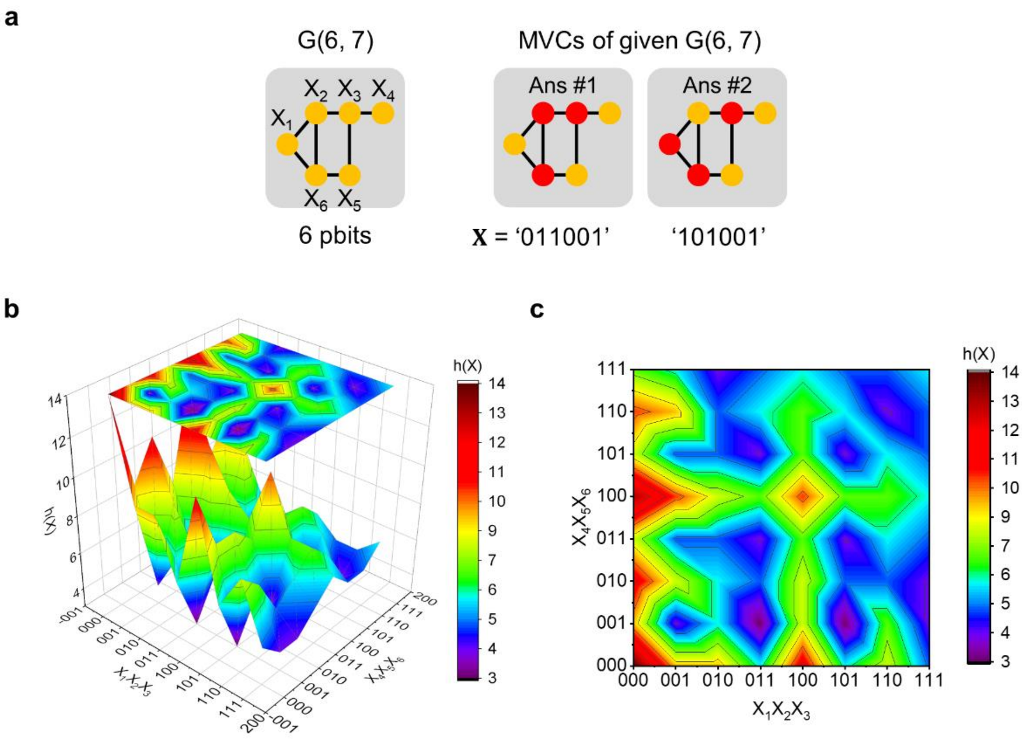

Fig. S9. a Given graph G(6, 7) and its MVCs. **b, c** Energy maps for every states (**X**) based on defined Hamiltonian (Equation 10 in main text) to solving MVC problem.

Fig. S9a shows the minimal vertex coverings (MVCs) of the G(6, 7) graph presented in the main text. Given the relatively small scale of the problem, it is feasible to identify the correct (**X** = '011001' and '101001') and incorrect solutions using a brute-force algorithm.

The energy maps according to the Hamiltonian (Equation 10) is shown in Figs. S9b-c. This is implemented in the MBM, employing the Ising model approach[5]. The energy map reveals that the MBM system assigns global minimum energy states to **X** = '011001' and '101001', the same as the ground truth from the brute-force algorithm. There are also local minimum states, such as '010111', '011011', '011101', '101011', '101101', '111110', '111001', and '111010’, which are energetically favorable compared to their immediate surroundings but hold higher energy than the global minimum states.

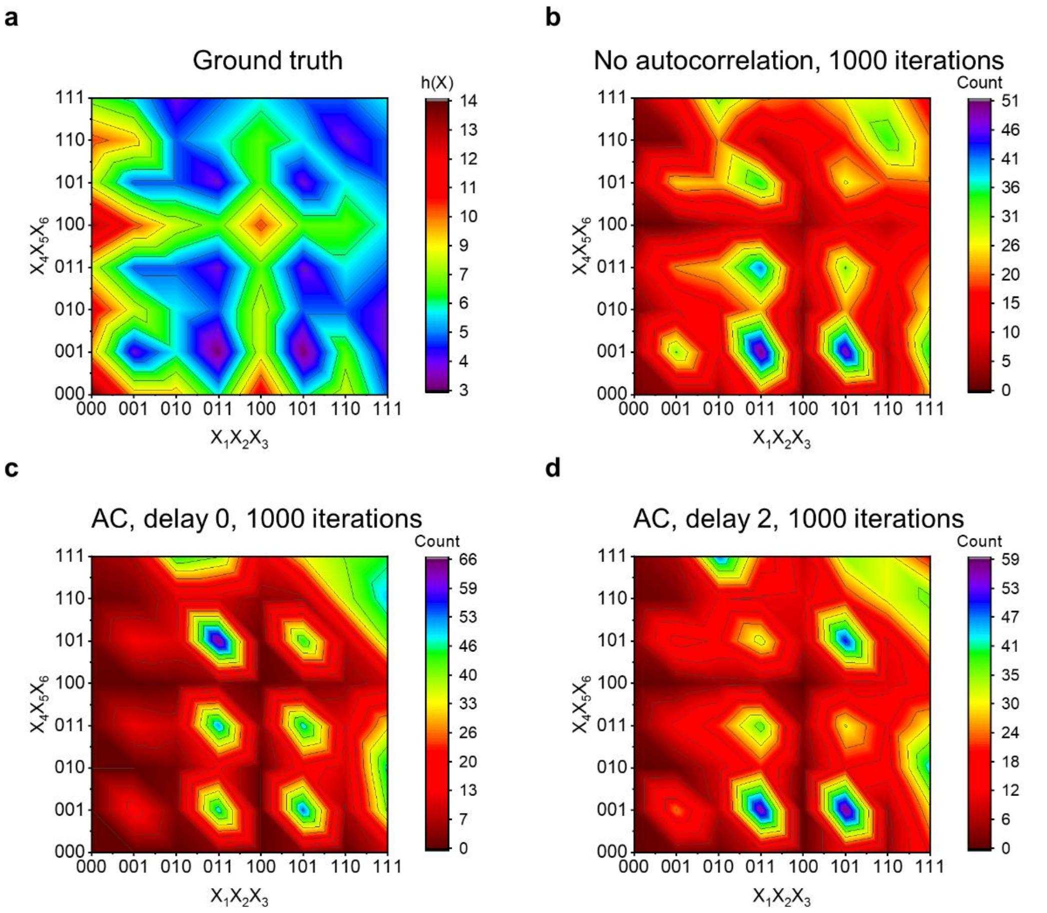

Fig. S10. a Energy map of MVC problem for given graph G(6, 7). MBM simulation results for every **X** states at 1,000 iterations in case of **b** un-autocorrelated, **c** autocorrelated, no delay period (Δ), **d** autocorrelated, $\Delta = 2$.

In accordance with the Boltzmann law, global minimum states appear most frequently, followed by the local minimum states. Thus, we anticipated that $\bar{Y}_{\text{incor}}$, which represents the average occurrence of the five most frequently appearing non-correct cases, would primarily consist of these local minimum states. Figs. S10b-d validate this assumption by displaying the MBM simulation results for every configuration **X** in both the presence and absence of autocorrelation.

Autocorrelation-induced non-independent probabilistic behavior hinders the MBM from effectively exploring the solution space represented by the energy map, leading to an increase in occurrence of local minimum states. Consequently, we observe this effect as a rise in $\bar{Y}_{\text{incor}}$ in Figs. S10c-d as well as Figs. 4c-d in the main text. More specifically, as shown in Fig. S10c, without the inclusion of a delay period, the '011101' configuration becomes most prominent in the presence of autocorrelation. This highlights the risk that autocorrelation could cause a local minimum state to appear dominantly within a small number of iterations, thereby deviating from the ground truth.

13. Experiment on probabilistic oscillation in NbO_x oscillator fabricated with different condition

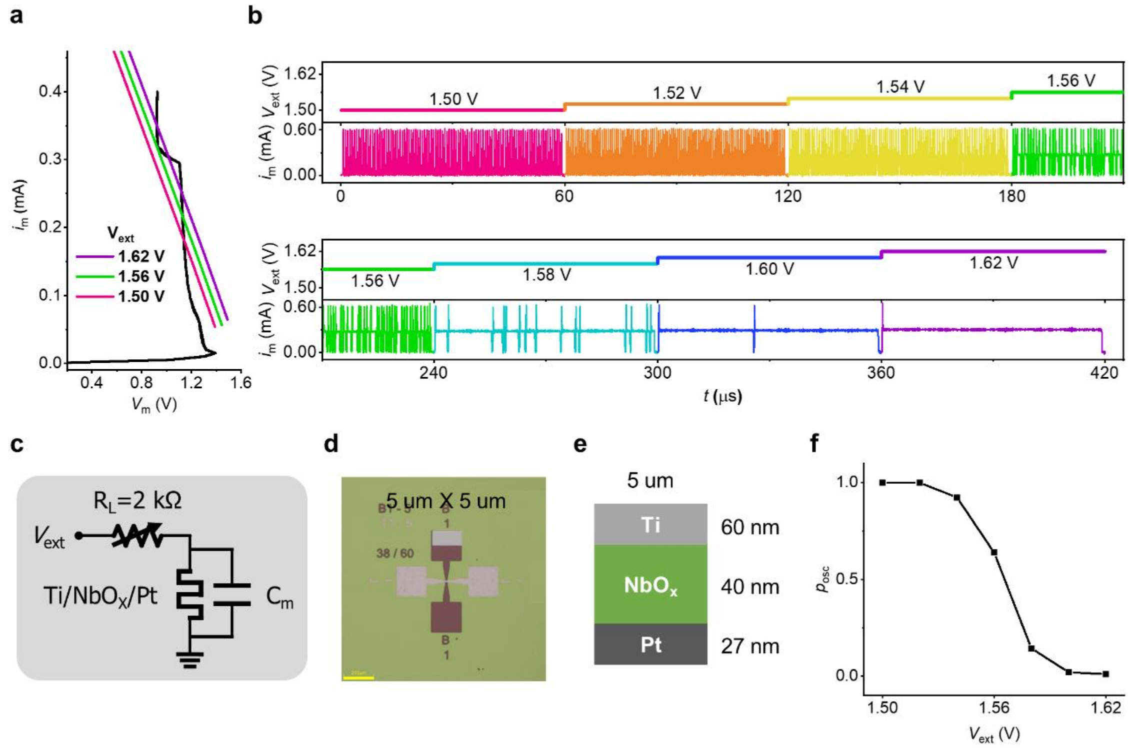

Fig. S11. Probabilistic oscillation in Ti/NbO_x/Pt volatile memristor a Threshold switching *I*-*V* curve of Ti/NbO_x/Pt volatile memristor (black) and the load lines of 2.0 kΩ series resistor under various V_{ext}. **b** Probabilistic current oscillations by ramping the V_{ext} from 1.50 V to 1.62 V at 0.02 V interval. **c** Self-oscillator circuit consists of a memristor, series resistor, and internal capacitive volume of the NbO_x layer. **d** Optical microscope image of memristor used in this experiment and **e** schematic of Ti/NbO_x/Pt structure with each layer's thickness. **f** Oscillation probability distribution to the V_{ext} showing sigmoidal decreases to the V_{ext}.

Fig. S11 shows the probabilistic oscillations of an NbO_x memristor-based oscillator fabricated under different conditions compared to the device described in the main text. For this device, the dimensions are 5 x 5 μm^2, featuring a Pt bottom electrode and a Ti top electrode. A 40 nm thick NbO_x layer was deposited using the reactive sputtering method, maintaining an Ar:O_2 ratio of 13:7.

References

1. Khovanov, I. A., Polovinkin, A. V., Luchinsky, D. G. & McClintock, P. V. E. Noise-induced escape in an excitable system. *Phys. Rev. E* **87**, 032116 (2013).

2. Yamakou, M. E., Tran, T. D., Duc, L. H. & Jost, J. The stochastic Fitzhugh–Nagumo neuron model in the excitable regime embeds a leaky integrate-and-fire model. *J. Math. Biol.* **79**, 509–532 (2019).

3. Hull, J. C. *Options, Futures, and Other Derivatives*. (Pearson, 2022).

4. Huang, S. T. & Cambanis, S. Stochastic and Multiple Wiener Integrals for Gaussian Processes. *Ann. Probab.* **6**, 585–614 (1978).

5. Lucas, A. Ising formulations of many NP problems. *Front. Phys.* **2**, 5 (2014).

6. Hertz, J. *et al.* The Hopfield Model. *Introd. to Theory Neural Comput.* 11–41 (2018).
